# Acyl peptide hydrolase degrades monomeric and oligomeric amyloid-beta peptide

**DOI:** 10.1186/1750-1326-4-33

**Published:** 2009-07-23

**Authors:** Rina Yamin, Cheng Zhao, Peter B O'Connor, Ann C McKee, Carmela R Abraham

**Affiliations:** 1 Department of Biochemistry, Boston University School of Medicine, 72 East Concord Street, Boston, K620, MA 02118, USA; 2 Mass Spectrometry Resource, Boston University School of Medicine, 670 Albany Street, Boston, MA 02118, USA; 3 Department of Neurology, Boston University School of Medicine, 72 East Concord Street, Boston, MA 02118, USA; 4 Department of Pathology, Boston University School of Medicine, 72 East Concord Street, Boston, MA 02118, USA; 5 Geriatric Research Educational and Clinical Center, Bedford Veterans Administration Medical Center, MA 01730, USA; 6 Department of Medicine, the, Boston University School of Medicine, 72 East Concord Street, Boston, MA 02118, USA

## Abstract

**Background:**

The abnormal accumulation of amyloid-beta peptide is believed to cause malfunctioning of neurons in the Alzheimer's disease brain. Amyloid-beta exists in different assembly forms in the aging mammalian brain including monomers, oligomers, and aggregates, and in senile plaques, fibrils. Recent findings suggest that soluble amyloid-beta oligomers may represent the primary pathological species in Alzheimer's disease and the most toxic form that impairs synaptic and thus neuronal function. We previously reported the isolation of a novel amyloid-beta-degrading enzyme, acyl peptide hydrolase, a serine protease that degrades amyloid-beta, and is different in structure and activity from other amyloid-beta-degrading enzymes.

**Results:**

Here we report the further characterization of acyl peptide hydrolase activity using mass spectrometry. Acyl peptide hydrolase cleaves the amyloid-beta peptide at amino acids 13, 14 and 19. In addition, by real-time PCR we found elevated acyl peptide hydrolase expression in brain areas rich in amyloid plaques suggesting that this enzyme's levels are responsive to increases in amyloid-beta levels. Lastly, tissue culture experiments using transfected CHO cells expressing APP751 bearing the V717F mutation indicate that acyl peptide hydrolase preferentially degrades dimeric and trimeric forms of amyloid-beta.

**Conclusion:**

These data suggest that acyl peptide hydrolase is involved in the degradation of oligomeric amyloid-beta, an activity that, if induced, might present a new tool for therapy aimed at reducing neurodegeneration in the Alzheimer's brain.

## Background

Alzheimer's disease (AD) is a neurodegenerative disorder resulting from the pathological processing of APP that leads to the accumulation of amyloid-beta (Aβ) peptide, neuronal loss, synaptic dysfunction, inappropriate levels of neurotransmitters, and finally, to irreversible and progressive memory loss. Hallmark neuropathological features, namely senile plaques and neurofibrillary tangles, are found in the AD brain [1,2].

Cognitive decline in AD results from progressive dysfunction of synaptic connections within cortical neuronal microcircuits. Extracellular plaques were thought to be responsible for synaptic dysfunction and neuronal loss based on *in vitro *studies using synthetic Aβ fibrils which showed that fibrillar Aβ is toxic to neurons [3-5]. However, growing evidence suggests that this impairment is caused by accumulation of non-fibrillar, soluble oligomeric forms of the Aβ peptide, a process that triggers early synaptic disorganization prior to widespread synaptic loss and neurodegeneration [6]. Thus, soluble Aβ oligomers and probably the Aβ dimers represent the primary pathological species in AD [7,8]. Although fibrillar amyloid plaques and soluble Aβ oligomers exist in the brain at the same time, only a relatively weak correlation was found between fibrillar plaque density and the degree of dementia [9,10]. Meanwhile, levels of total soluble Aβ, and soluble Aβ oligomers correlated with the degree of synaptic loss and the severity of cognitive impairment [11,12]. Altered metabolic balance between Aβ generation and Aβ degradation likely results in Aβ accumulation and in the change from Aβ monomers to the toxic forms of Aβ oligomers. It is critical, therefore, to find ways to prevent Aβ oligomer accumulation in order to prevent neuronal toxicity and the cascade into neurodegeneration [13].

One way to reduce Aβ oligomer accumulation in the brain is by increasing the activity of the Aβ-degrading enzymes. Several proteases were reported as capable of degrading the Aβ peptide. Neprilysin (NEP) [14], and Insulin Degrading Enzyme (IDE) [15], were proven to be effective *in vitro *and *in vivo *in decreasing Aβ levels in mice brains. These proteases are not specific to Aβ, as IDE is known to affect insulin and beta-endorphin [16], and neprilysin cleaves a variety of physiologically active peptides such as substance P, bradykinin, oxytocin, Leu- and Met- enkephalins, neurotensin, and more [17]. If these enzymes were to be used as targets for treating AD, they would need to be activated in a way that selectively increases their Aβ-degrading activity while minimally affecting their activity on other substrates [18,19].

We recently purified to homogeneity, as judged by a single band on a silver stained gel, and identified by mass spectrometry, the serine protease acyl peptide hydrolase (APEH) as an enzyme that degrades Aβ in SKNMC neuroblastoma cells [20]. The precise biological activity of APEH is unknown [21]. APEH is a 75–80 kDa peptidase and one of four members of the prolyl oligopeptidase family of serine proteases. APEH degrades peptides between 30 and 50 amino acids long and preferentially degrades oxidized peptides [22]. The enzyme is expressed in heart, liver, kidney, testis, brain, and also found in erythrocytes, and plasma [21]. Shimizu and colleagues reported APEH mediated degradation by APEH of oxidized peptides *in vitro *and demonstrated that APEH activity was protective to cells under oxidative stress conditions by clearance of denatured proteins in coordination with the proteasome system [23,24]. However, other studies, using APEH isolated from the porcine gastro-intestinal tract, porcine liver, or from human erythrocytes, describe APEH as an enzyme that removes acyl groups from N-acylated peptides [25-27].

We are the first to isolate APEH as an Aβ-degrading enzyme and here show by mass spectrometry that APEH cleaves Aβ at amino acids 13, 14 and 19. In addition, we found elevated APEH expression in brain areas that are rich in Aβ plaques suggesting that APEH levels are responsive to increases in Aβ levels. Lastly, our results from tissue culture experiments using transfected CHO cells expressing APP751 bearing the V717F mutant indicate that APEH preferentially degrades dimeric and trimeric Aβ, the most toxic forms of Aβ shown to affect LTP, and thus memory. These data indicate that if proven *in vivo*, APEH may serve as a new therapeutic target for AD and that induction of its activity in the brain can lead to prevention or reduction in Aβ accumulation and Aβ oligomers formation in AD.

## Results

### APEH cleaves Aβ monomers at three sites

**Figure 1:**
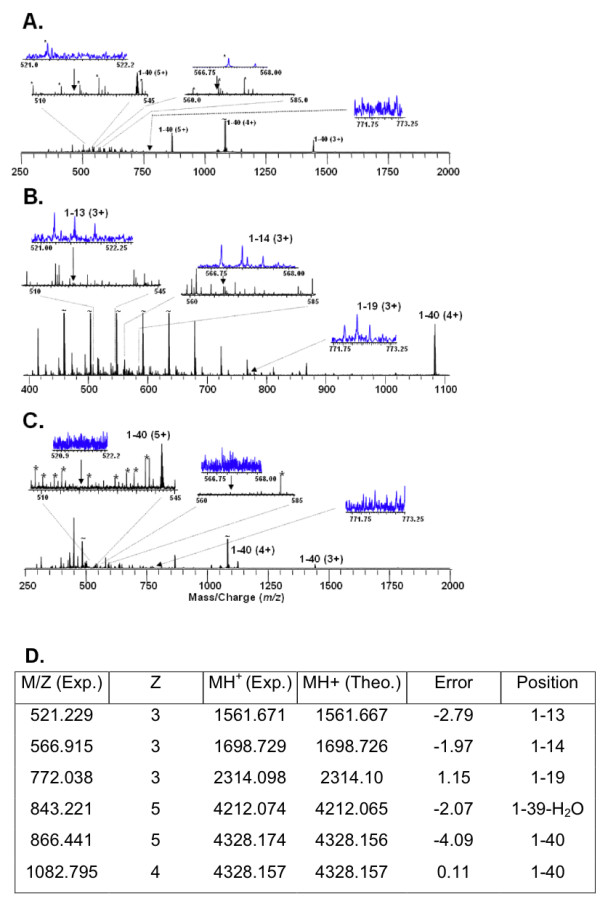
**Mass spectrometry analysis of soluble Aβ_1–40 _digested by APEH**. All three samples were incubated simultaneously for 5 hours at 37°C. A) The ESI-FTICR mass spectrum of Aβ_1–40_alone. B) The ESI-FTICR mass spectrum of Aβ_1–40 _incubated with the APEH enzyme. C) The ESI-FTICR mass spectrum of Aβ_1–40_incubated with both the APEH enzyme and the APEH specific inhibitor Ac-Leu-CK. The m/z regions at 522, 567, and 772 are expanded in the insets and correlate within 3 ppm with the calculated masses for the peptide fragment ions [1–13]^3+^, [1–14]^3+^, and [1–19]^3+ ^respectively. D) Observed (Exp.) and calculated (Theo.) masses of the peaks observed in A-C.

Our previous manuscript [20] described the isolation and identification of the serine protease acyl peptide hydrolase as an enzyme that degrades Aβ. In order to determine the exact cleavage sites in the Aβ peptide, the APEH enzyme that was purified from transfected cells was incubated with synthetic Aβ 1–40 for 5 hours. C18 ziptip desalting was performed followed by direct injection electrospray FTICR mass spectrometry on the resulting peptide fragment mixture. Samples were "freshly" analyzed every time in order to minimize the loss of hydrophobic Aβ peptide and its fragments. Figure 1 represents the results of these experiments on three peptides incubated simultaneously as described above. Figure 1A shows the Aβ peptide alone; Figure 1B shows the Aβ peptide incubated with the APEH enzyme, and Figure 1C shows the Aβ peptide incubated with the APEH enzyme and its specific inhibitor. The insets in Figures 1A–C show the m/z regions 522, 567, and 772, corresponding to the [1–13]^3+^, [1–14]^3+^, and [1–19]^3+ ^ion masses. These mass assignments are within 3 ppm of the expected mass as shown in the table (Figure 1D). Therefore cleavage of Aβ by APEH occurs after amino acids 13, 14 and 19. While the samples were reacted with APEH for 5, 8, and 24 hours, the signal intensities were not consistent except for the 5-hour time point shown in Figure 1. Thus, these data clearly show the presence of these fragments, it does not (and can not) say anything else about the reaction and particularly does not yield any kinetic data or information about secondary fragmentation. With such an experiment, it is not possible to predict, a priori, that the signal intensity of fragments would increase or decrease monotonically with time because, in addition to these peptides being generated by the APEH, the Aβ is slowly being lost to precipitation or by "sticking" to the side of the tubes resulting in an undetectable amount of fragments left in the tube.

**Figure 2:**
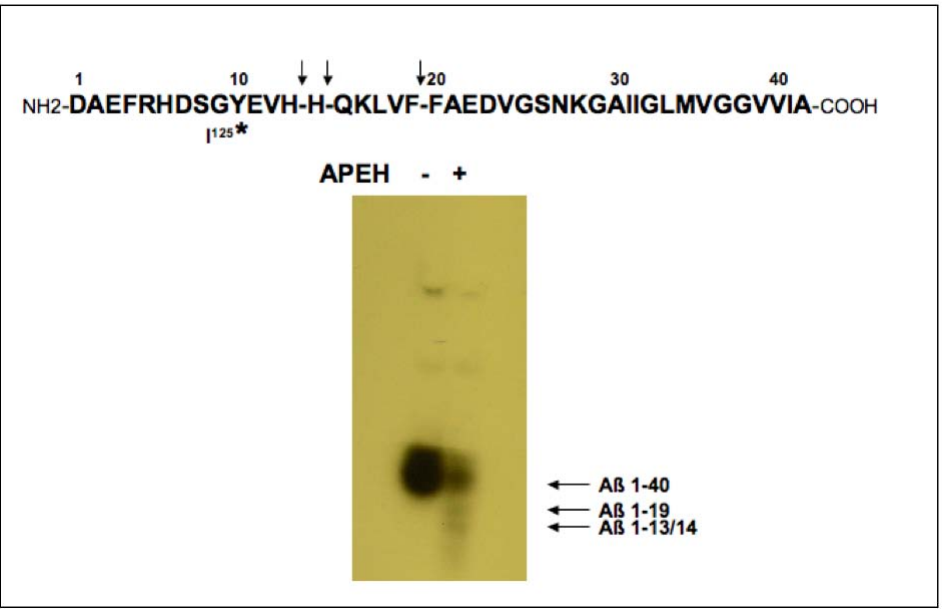
**I ^125 ^– Aβ fragments following APEH cleavage**. Soluble Aβ peptide iodinated on amino acid 10 was incubated for 5 hours with or without purified APEH. Following incubation, samples were separated on a 10–20% Tris-tricine gel. The gel was then dried and exposed to film. Full length uncut Aβ fragment, as well as the cleaved Aβ fragments containing the radiolabeled Lys^10 ^are seen on the autoradiograph. Above the autoradiograph is the amino acid sequence of the Aβ peptide. * denotes iodinated amino acid. Arrows indicate APEH cleavage sites.

To confirm this result, the same purified enzyme was incubated with iodinated Aβ1–40 peptide and the cleaved fragments were separated on an acid/urea gel. The autoradiograph in Figure 2 shows the radioactively labeled fragments resulting from cleavage by APEH. The synthetic Aβ peptide used in this experiment was iodinated on amino acid 10 (Tyrosine) therefore only the fragments containing the iodinated tyrosine are seen on the gel: *i.e. *the 1–13, the 1–14 and the 1–19 fragments. In addition, the full length Aβ peptide band can be seen. These results are indicative of the cleavages seen by the mass spectrometry analysis.

### APEH transfection reduces the amount of Aβ monomers, dimers and trimers in conditioned medium of 7PA2 cells

**Figure 3:**
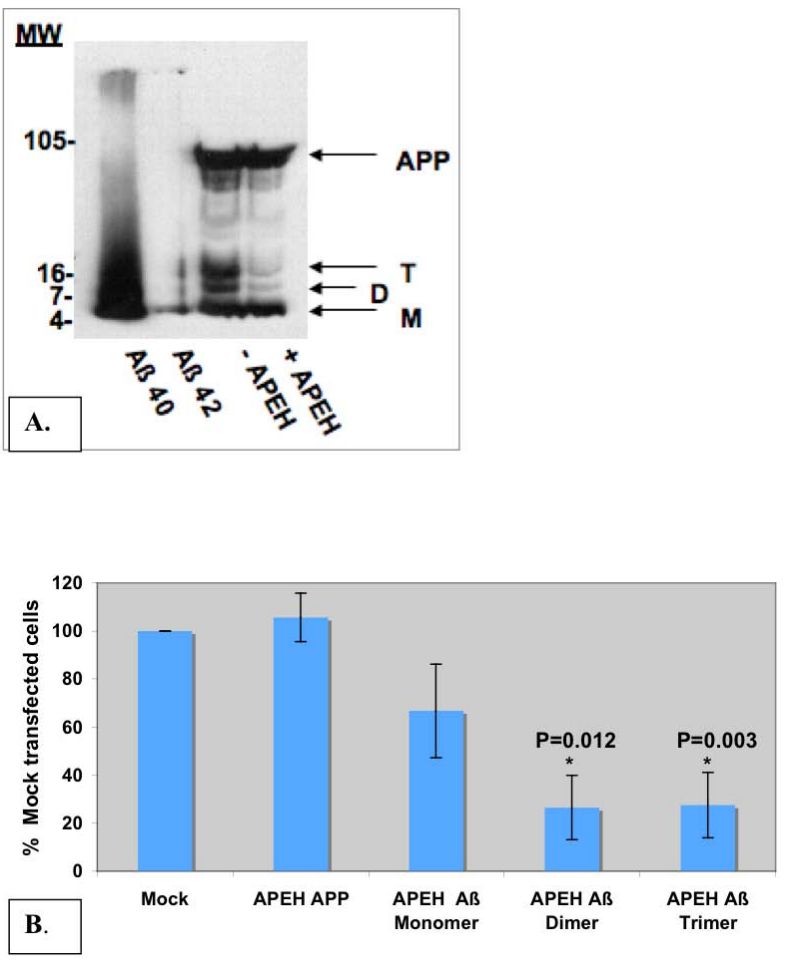
**Levels of Aβ oligomers in conditioned medium of 7PA2 cells after transfection with APEH**. Serum-free conditioned media were collected from 7PA2 cells that were non-transfected or transfected with APEH (lanes 3&4). Aβ was immunoprecipitated using a mixture of 6E10 and 4G8 antibodies. Samples were then separated on a 16% Tris-tricine gel and western blot was probed with 6E10 antibodiesA Representative gel out of four experiments. Lanes 1) synthetic Aβ 1–40 peptide; 2) Synthetic Aβ 1–42; 3) IP from cells not transfected with APEH; 4) IP from cells transfected with APEH. Arrows indicate: secreted APP (APP) as well as monomers (M), dimers (D) and trimers (T) forms of the Aβ peptideB Diagram indicating the average levels of Aβ in the presence or absence of APEH from four independent experiments. Values are presented as % of Mock for each Aβ form as follows: mock = 100% (n = 4); APEH APP band (n = 2); APEH Aβ Trimer (n = 3); APEH Aβ Dimer (n = 3); APEH Aβ Mono (n = 4). * Indicates statistically significant p value determined by T-test.

In order to determine whether APEH can cleave Aβ oligomers, in addition to monomers, we used 7PA2 cells that secrete oligomeric forms of Aβ into their conditioned medium [28,29]. Following transfection of 7PA2 cells with APEH cDNA, serum free conditioned medium was collected from the cells. Aβ was immunoprecipitated from the conditioned medium using 6E10 antibodies. Western blot of Aβ and its fragments with or without APEH overexpression is shown in figure 3. A clear reduction in both dimeric and trimeric forms of Aβ is demonstrated in the cells overexpressing APEH, while there is no difference in the secreted APPα. The reduction in dimeric and trimeric Aβ was significant and reproducible, however, there was variability in the reduction of the monomeric Aβ band between experiments. A representative experiment is shown in figure 3A. Figure 3B is a graph representing an average of four independent experiments.

### APEH relative expression in brain tissue samples

**Figure 4:**
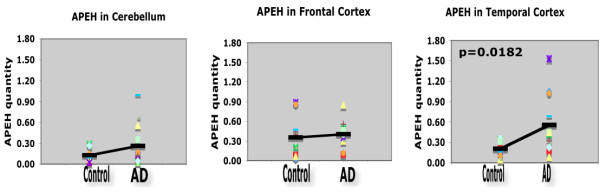
**Relative expression of APEH in human brain samples**. Relative expression of APEH was obtained by Real-Time PCR on mRNA from 12 AD and 9 age-matched control brain samples. mRNA was prepared from cerebellum, frontal cortex, and temporal cortex from each individual. APEH quantity was normalized to β-actin in each sample, and the p values were determined by student T-test and by Mann-Witney nonparametric rank test.

We previously reported the results of a pilot study done using real-time PCR on 6 AD and 6 age-matched controls samples from the frontal lobe [20]. In that previous report the sample size was small and the difference in APEH expression was not statistically significant. Here, we extended our study in order to determine whether there is a correlation between APEH expression levels and plaque load in AD brain samples. We performed real-time PCR experiments on 12 AD and 9 age-matched control brain samples. For each individual sample we determined the APEH relative expression in three areas of the brain: the cerebellum which is known to be low in plaque load in AD, and the frontal and temporal cortex both of which are known to be rich in plaques. The results of this study are described in figure 4 and in table 1. As seen in table 1, on a scale of 1–8 the plaque load in the cerebellum is between 0–1 in both AD and controls. On the other hand, the plaque load in the frontal and temporal cortices of the AD samples is significantly higher (average 7–8) than the controls (average 1). As for APEH relative expression, in the cerebellum we found no significant difference between AD (average 0.262) and age matched controls (average 0.125). We also found no significant difference in APEH relative expression between AD (average 0.35) and controls (average 0.4) in the frontal cortex. However, in the temporal cortex the APEH relative expression is significantly increased in AD compared to controls (0.55 in AD vs. 0.2 in controls). Furthermore, APEH relative expression, in both the frontal and temporal cortices, is two times higher than that of the cerebellum (0.35–0.4 as opposed to 0.1–0.2). The results of this study indicate that more APEH enzyme may be produced in areas where Aβ levels are higher. This observation fits data reported by others of lower Aβ levels in cerebellum as compared with frontal and temporal cortices in AD patients and in animal models [30,31].

**Table 1:** Summary of age, gender, PMI and Aβ load in the AD and age-matched controls brain tissues.

**Sample**	**Age (years)**	**Gender (M/F)**	**PMI (hrs)**	**Aβ Load** **(scale 0–8)**		
**AD**				**C**	**F**	**T**

1	74	M	N/A	2	8	8

2	72	M	18	0	8	8

3	87	M	4	0	4	6

4	83	M	5	0	8	8

5	84	M	2	1	4	4

6	88	M	N/A	0	8	8

7	72	M	2	3	8	8

8	79	M	23	0	8	8

9	92	M	10	0	6	6

10	82	F	4	2	8	8

11	83	F	2	2	7	7

12	85	F	3	0	7	5

Average	81.7 +/- 6.3		7.3	0.8	7	7

**Controls**						

1	67	M	3	0	0	0

2	74	M	N/A	0	2	0

3	87	F	24	0	3	3

4	62	M	N/A	0	0	0

5	89	M	6	0	0	0

6	85	M	48	0	4	3

7	87	F	4	0	0	0

8	78	F	6	0	4	3

9	87	M	12	0	2	0

Average	79.5 +/- 9.9		14.7	0	1.7	1

Table summarizes the post mortem intervals (PMI) and the Aβ load for each brain tissue specimen used. The plaque counts were performed in specific areas using semi-quantitative measures generated using CERAD methodology. They are purely estimates of pathology on a scale of 0–8. There was no statistically significant difference in age between groups by T-test. In the AD group, the majority is males since the samples were obtained from a VA hospital.

C = Cerebellum; F = Frontal cortex; T = Temporal cortex; Gender: M = Male; F = Female; PMI = post mortem interval.

## Discussion

Progressive accumulation of the Aβ peptide in brain regions serving memory and cognition is an early and invariant feature of Alzheimer's disease [32]. Therefore, understanding of the mechanism that controls Aβ degradation and finding ways to prevent its accumulation in the AD brain will serve as an effective therapeutic target for slowing the progression of the disease. The best studied enzymes capable of degrading the Aβ peptide, NEP [14], IDE [15], plasmin [33], and endothelin converting enzyme (ECE-1) [34] originate from a variety of cell types, degrade Aβ of various conformational states and in different subcellular locations. However, these enzymes have other substrates in addition to Aβ. We were the first to describe APEH as an enzyme that degrades Aβ [20]. To date, no other known biological substrates were reported for APEH, suggesting that Aβ could be its major substrate in the brain.

We have previously shown that the Aβ-degrading activity of APEH is inhibited by APEH specific inhibitors, as well as by synthetic Aβ peptide in a dose dependent manner [20]. Using mass spectrometry we show here that APEH cleaves Aβ at amino acids 13, 14 and 19. We also confirm this finding by gel electrophoresis of iodinated Aβ, as shown in figure 2.

As for APEH expression levels, our earlier real-time PCR data indicated that APEH expression in AD brains is five times lower than in age-matched controls, but the results obtained from 6 AD and 6 age-matched control samples were not statistically significant [20]. However, in this expanded study that includes a larger number of samples and three different areas of the brain from each patient, we show that APEH expression is elevated in brain areas that are rich in Aβ plaques (figure 4 and table 1). The controversial results could arise from the small number of samples used in the previous study. Samples from the temporal and frontal cortices of AD patients showed similar amount of plaque load and higher levels of APEH relative expression as compared with cerebellum samples of AD, an area known to contain few or no plaques. This result suggests that APEH levels are responsive to increases in Aβ levels. In studies examining the Aβ distribution in different areas of the brain, cerebellum has a low amount of Aβ and also less amyloid plaques in AD patients compared to temporal and frontal cortices that have the highest Aβ levels and the highest number of plaques [30,31]. It seems that APEH levels increase in areas where Aβ levels increases as a biological response to rid the excess Aβ as seen in figure 4. Higher APEH levels are seen in both AD and controls in frontal and temporal cortices compared to cerebellum, plus a significant increase is observed in temporal cortex of AD compared to controls.

It has been shown in animal models that reduction in both neprilysin and IDE expression in the brain correlated with increased Aβ dimers and an increased plaque load. Furthermore, in mice having both copies of NEP genes silenced, the loss of function lead to an elevation in the brain and in plasma of Aβ 40 and 42, prolonged the half life of soluble Aβ in brain interstitial fluid, and markedly increased hippocampal amyloid plaque burden [35-37]. Reduction in hippocampal IDE protein levels were reported and were associated with the APOE-4 allele presence in the AD patients [38]. Similarly, by western blot and by PCR experiments using reverse transcriptase, NEP protein and mRNA levels, respectively, were found to be lowest in AD patients in the hippocampus and temporal gyrus, two areas that are vulnerable to senile plaque development, and found to be highest in caudate and peripheral organs which are resistant to Aβ plaque deposition [39]. It is possible that the loss of IDE and neprilysin in AD brain is due to the loss of neurons that produce these two enzymes or the hypofunction of live neurons as it has been reported for choline acetyltransferase (ChAT) in AD. While the upregulation in APEH could come from its overexpression in astrocytes or microglia, which are known to be activated both in AD and in brains of animal models of AD. Once expression of IDE and NEP is reduced in the AD brain leading to Aβ accumulation, APEH expression levels in frontal and temporal cortex may increase in order to remove excess Aβ. The difference in APEH expression levels between AD and controls are significant only in the temporal cortex, yet the plaque load is high in both the frontal and temporal cortex. This result is consistent with the concept that aggregated Aβ in plaques may not reflect a direct correlation with APEH as seen with other measures of pathology or behavior in AD animal models. Thus, APEH levels may be better correlated with soluble Aβ [20]. In addition, as seen in table 1, there is a difference in PMI between the AD and control samples used in the experiment (control PMI being longer than the AD PMI). This difference may contribute to the fact that the increasing trend in APEH level between cerebellum and the frontal cortex was not statistically significant, while the difference in APEH levels between control and AD in temporal cortex is statistically significant. It is possible that if there was no difference in PMI between the AD and control samples, the APEH levels could be more accurately attributed to difference in levels of Aβ peptide monomers in these two areas of the brain. As it is clear from the data presented that aggregated Aβ load (plaque load) is similar in frontal and temporal cortices despite the difference in PMI. Therefore we have to assume that APEH levels are responsive to increase in soluble monomeric or oligomeric Aβ levels and this may occur before the insoluble plaques are formed.

This responsive effect of APEH to increasing Aβ levels needs to be further elucidated *in vivo*, in transgenic mice in order to determine the importance of APEH as an Aβ-degrading enzyme *in vivo*.

Lastly, our tissue culture experiments using transfected CHO cells expressing APP751 bearing the V717F mutation indicate that APEH preferentially degrades dimeric and trimeric forms of Aβ, the most toxic forms. These oligomers, and specifically the Aβ dimers, were shown to block LTP in animal models and disrupt memory of learned behavior in normal rats [8], and thus probably affect memory in the AD patients. We show here that 7PA2 cells overexpressing APEH have less monomeric, dimeric and trimeric forms of Aβ in their conditioned medium. This result indicates that APEH degrades not only monomers but also soluble dimers and trimers forms of Aβ oligomers. It is important to note that due to the fact that no antibodies are available at this time for the enzyme APEH, the only way we could purify the enzyme for the purpose of these experiments was by using the ProBond purification system which yields a very limited amount of active enzyme from transfected cells. Therefore it was not possible for us to perform additional experiments using purified enzyme with purified Aβ oligomers, which could be a more direct proof for such an activity. By over expressing APEH in the cells we mimic the "in vivo" conditions. We therefore recognize that these experiments are not a solid proof that APEH preferably degrades the oligomers, but the clear difference between the "with/without" APEH conditions shown, indicate this possibility. The details of the exact mechanism should be further investigated and proved by testing the relevance of APEH in Aβ degradation in mice models by using either APEH knock-out or overexpressing mice. It is possible that APEH activity becomes more significant as the Aβ oligomers start to form. Although APEH is a cytosolic enzyme, it is also found secreted as we have shown [20] and in this manuscript. If its role is proven in animal models, APEH can serve as an important therapeutic target for slowing the Aβ induced neurodegeneration and dementia in AD.

## Conclusion

The findings presented here show that the enzyme APEH cleaves Aβ at amino acids 13,14 and 19 and that it cleaves preferentially Aβ dimers and trimers in addition to Aβ monomers in vitro and in cell culture, respectively. Real-time PCR data presented here show that APEH levels in different areas of the brain are responsive to increase in Aβ levels. We hypothesize that APEH activity increases as result of the brain response to increasing levels of Aβ in these areas. In order to prove our hypothesis further experiments in APEH transgenic mice need to be performed. In vivo experiments will determine the significance of the Aβ-degrading activity of APEH compared to the other known Aβ-degrading enzymes such as NEP and IDE present in these same brain areas as well. If proven in animals to significantly slow down plaque formation, APEH can become a new therapeutic target for AD.

## Methods

### Mass spectrometry experiments and sample preparation

COS 7 cells were transfected with APEH cDNA tagged with His and V5 and allowed to recover for 24–48 hours prior to collection of cell lysates. The APEH enzyme was purified from the transfected cells using the ProBond kit (Invitrogen) under native conditions as described previously [20]. Samples were separated on NuPAGE gels (Invitrogen) and the enzyme activity in 10 μL of each purified fraction was determined by activity assay on the APEH specific chromogenic substrate AANA (Bachem), as described in [20]. All the active fractions that showed a single APEH band on the silver stained gel, were pooled together and dialyzed against 50 mM Tris buffer in order to remove the imidazole that was used to elute the samples from the Pro-Bond column. Following dialysis, the pooled fractions were concentrated by filtration through a Centricon 3 filter (Amicon) and enzyme activity was assayed again on the AANA substrate. Since the amount of protein in the purified fractions was below detectable levels by silver staining of gels, we performed all the purifications under the same conditions, and after concentrating the sample 4× we used 50 μL of the purified enzyme fraction for mass spectrometry. Fifty micro-liters of purified enzyme were incubated with 50–100 pmole of freshly dissolved (in 50 mM Tris-HCl pH = 7.4) synthetic Aβ1–40 peptide (Bachem) in presence or absence of the APEH specific inhibitor Ac-Leu-CK. Samples were incubated for 5, 8 or 24 hours at 37°C water bath and immediately following incubation were analyzed by mass spectrometry. In some experiments (where indicated) synthetic Aβ 1–40 peptide iodinated at position 10 (Tyrosine) was used. The iodinated Aβ was purchased lyophilized from Peninsula Laboratories and reconstituted in water immediately before use.

### Mass spectrometry analysis

After C18 ziptip (Millipore) desalting, untreated Aβ (1–40), Aβ (1–40) incubated with APEH, and Aβ (1–40) incubated with both APEH and the inhibitor Ac-Leu-CK, were monitored by electrospray mass spectrometry, using a custom built ESI-qQq-FTMS [40,41] in direct injection mode. Figure 1 shows the resulting mass spectra, discussed below. The spectrum clearly shows the 3+, 4+, and 5+ charge states of the 1–40 as well as a small amount of a dehydrated 1–39 contaminant. The *m/z *regions at 521, 567, and 772 are expanded in the insets for comparison. There is substantial "chemical noise" below *m/z *700, which is due to buffer contaminants in the sample, which are common in direct injection ESI mass spectra. While these could, perhaps be removed by HPLC or solid phase microextraction [42] this approach was avoided due to potential for loss of the highly hydrophobic Aβ and its fragments.

### Viewing the Aβ cleaved fragments on acid/urea gels

Gradient acid/urea gels (10–20% acrylamide) were prepared and pre run anode to cathode at 250 v for 30 minutes at 4°C in 5% glacial acetic acid running buffer. Samples were prepared with 6× sample buffer containing 80% formic acid, 60% sucrose, and 0.02% methyl green. Pharmacia protein molecular weight markers, range 2,512–16,949 were dissolved in PBS and 10 μL aliquots were frozen in -20°C. When needed, each aliquot was diluted in the 6× sample buffer. Following the pre run, samples were loaded on the gel and gel was run from anode to cathode in the cold room (4°C) with increasing the voltage every 3 minutes as follows: 25 volts, 50, 100, 200 and then 275 volts till the end of the run. The gels were run until the dye ran off the bottom of the gel. Before transfer to nitrocellulose the acid/urea gels were neutralized in a washing glass tray with 200 mL of Tris/glycine transfer buffer on a rocker for 15 minutes. This wash was repeated for a total of 5 times. After the neutralization washes, regular western blot gel transfer was performed for 2.5 hours (100 V) at 4°C. Before probing with antibodies, the MW marker lane was cut out and stained with Ponceau S and the markers bands were marked with a pen before destaining with tap water. The rest of the membrane was boiled in PBS for 5 minutes in a glass dish and was cooled down in PBS before blocking with 5% milk in TBST consisting of 20 mM Tris-HCl, 150 mM NaCl, pH = 7.4 containing 1% Triton X-100 for 45–60 minutes. The membrane was incubated with primary anti Aβ monoclonal antibody 6E10 (1:1200 dilution) in 5% milk solution overnight on a rocker at 4°C. The following day the membrane was washed 3× with 1% BSA in TBST for 20 minutes each wash, and secondary antibody anti mouse HRP (1:6000 dilution) was incubated in 2.5% milk in TBST for 3 hours. After the secondary antibody, membranes were washed 3× for 15 minutes each in TBST, and placed in ECL solution (Pierce Super Signal West Pico) for 5 minutes prior to development. In some experiments, as indicated in figure legends, the Aβ fragments were run on 10–20% Tris-tricine gels. The western blots were done as described above.

### Transfection of 7PA2 cells

CHO cells stably transfected with the APP_751 _cDNA bearing the Val 717 Phe familial AD mutation (referred to as 7PA2 cells) [28,29] were obtained from Dr. D. Selkoe, Harvard Medical School. The 7PA2 cells were co-transfected with 5 μg of APEH cDNA using lipofectamine (Invitrogen). After transfection cells were allowed to recover 24 hours in growth medium containing 10% FBS. The next day, the cells were washed with serum free medium and incubated with 4.5 mL of serum free opti-MEM for 18 hours. The CM was then collected and the Aβ was immunoprecipitated (IP) using the 6E10 antibody (1:800 dilution). Pellets were run on 10–20% Tris-Tricine gels and transferred to a nitrocellulose membrane. Western blots were probed with 6E10 antibodies (1:1000) as described above. The different Aβ forms were detected after ECL development or autoradiography.

### RNA preparation from human brain tissues and real-time PCR

Frozen human brain tissues of cerebellum, frontal, and temporal cortices from each of 12 AD and 9 age-matched controls were obtained from the Boston University Alzheimer's Disease Center Brain Bank. mRNA was prepared from each sample using RNeasy (Qiagen) protocol. 2 μg mRNA from each sample was used for preparation of first strand cDNA. The detection of APEH relative expression was measured by real-time PCR as described in [20]. Results were normalized to β-actin. The P values were determined by student T-test.

## Abbreviations

Aβ: Amyloid beta; AD: Alzheimer's disease; APEH: Acyl peptide hydrolase; APP: Amyloid precursor protein; AANA: N-acetyl-alanyl-p-nitroanilide; CM: Conditioned medium; NEP: Neprilysin; IDE: Insulin degrading enzyme; TBST: Tris buffered saline plus Triton X-100.

## Competing interests

The authors declare that they have no competing interests.

## Authors' contributions

RY designed and carried out all the experiments, performed the statistical analysis and drafted the manuscript. CZ and PBO performed the mass spectrometry experiments and analysis. ACM supplied the human brain tissues, performed the Aβ load measurements and analysis, and participated in discussion and analysis of the real-time PCR data. CRA contributed to the design, discussion and data analysis of all the experiments, and edited the manuscript. All authors read and approved the final manuscript.
